# Risk factors and clinical consequences of interval cancers arising within faecal immunochemical testing-based colorectal cancer screening programme

**DOI:** 10.1093/bjsopen/zraf096

**Published:** 2025-10-08

**Authors:** Adam D Gerrard, Roberta Garau, Yasuko Maeda, Alastair Thomson, Evropi Theodoratou, Malcolm G Dunlop, Farhat V N Din

**Affiliations:** Cancer Research UK Scotland Centre, Institute of Genetics and Cancer, University of Edinburgh, Edinburgh, UK; Department of Colorectal Surgery, Western General Hospital, Edinburgh, UK; Cancer Research UK Scotland Centre, Institute of Genetics and Cancer, University of Edinburgh, Edinburgh, UK; Department of Colorectal Surgery, Western General Hospital, Edinburgh, UK; Cancer Research UK Scotland Centre, Institute of Genetics and Cancer, University of Edinburgh, Edinburgh, UK; Department of Surgery, Queen Elizabeth University Hospital, Glasgow, UK; Lothian Analytical Services, NHS Lothian, Edinburgh, UK; Cancer Research UK Scotland Centre, Institute of Genetics and Cancer, University of Edinburgh, Edinburgh, UK; Centre for Global Health, Usher Institute, University of Edinburgh, Edinburgh, UK; Cancer Research UK Scotland Centre, Institute of Genetics and Cancer, University of Edinburgh, Edinburgh, UK; UK Colon Cancer Genetics Group, Medical Research Council Human Genetics Unit, Medical Research Council Institute of Genetics and Cancer, Western General Hospital, University of Edinburgh, Edinburgh, UK; Cancer Research UK Scotland Centre, Institute of Genetics and Cancer, University of Edinburgh, Edinburgh, UK; Department of Colorectal Surgery, Western General Hospital, Edinburgh, UK

**Keywords:** colorectal cancer screening, faecal immunochemical testing, interval cancer, colorectal polyp, colorectal cancer

## Abstract

**Background:**

Colorectal cancer (CRC) screening programmes aim to detect early, asymptomatic cancers and improve survival. This study aimed to establish the proportion of interval cancers, and the consequences with regard to stage, clinical outcome, and overall survival. Risk factors associated with interval CRCs were investigated.

**Methods:**

The Scottish Bowel Screening Programme uses faecal immunochemical testing at a threshold of 80 µg haemoglobin per g as a positive trigger for investigation. Screening was offered to all eligible individuals in one region, from November 2017 to October 2021. Cancer registries were cross-checked to ensure complete capture of all cancers including interval CRCs. The primary outcome was rate of interval CRCs among participants with follow-up of 24 months, and its relationship to faecal immunochemical testing results, clinical variables, stage, time to diagnosis, and survival. The secondary outcome was identification of risk factors associated with interval CRCs.

**Results:**

The Scottish Bowel Screening Programme generated 316 583 tests during the study period. Participation was 71.0% of the eligible population (212 664 patients); it was greater among women (71.9 *versus* 70.0%; *P* < 0.001) and in higher socioeconomic areas (76.9 *versus* 58.6%; *P* < 0.001). In the screened population, 546 CRCs were diagnosed within 2 years of screening. Some 289 of these patients (52.9%) had positive bowel screening. There were 257 patients with interval CRCs, who waited a median of 13 (interquartile range 7–20) months for diagnosis. Of CRCs diagnosed, 24.9% had screening faecal immunochemical test results of < 10 µg haemoglobin per g. The interval CRC rate was greater in women, older patients, and among the least socioeconomically deprived. Interval CRCs were associated with worse 2-year all-cause mortality than screen-detected CRCs (23.0 *versus* 10.8%; *P* < 0.001). Importantly, 121 of the 257 interval CRCs (47.1%) had detectable faecal immunochemical test results at 10–79 µg haemoglobin per g.

**Conclusion:**

Patients with interval CRCs and a detectable faecal immunochemical test result below the predetermined threshold appear to be significantly disadvantaged with respect to stage at presentation and survival. Almost half of interval CRCs diagnosed within 2 years had detectable haemoglobin on screening faecal immunochemical test and would be a target for lower positivity thresholds.

## Teaser

Bowel cancer screening uses an initial faecal immunochemical test (FIT) with a positivity threshold set so that the colonoscopy workload generated is manageable. For people with negative bowel screening who were diagnosed with colorectal cancer (CRC) within 2 years (interval CRC), the median wait was 13 months for diagnosis. Women, older patients, and those from greater socioeconomic backgrounds had a higher interval CRC rate. Almost half of these interval CRCs had a detectable FIT level at screening but below the threshold (10–79 µg haemoglobin per g). All-cause mortality was significantly greater for interval CRCs compared with screen-detected lesions.

## Introduction

Colorectal cancer (CRC) is a global burden on healthcare, being the third most common cancer and second leading cause of cancer mortality^1^. There is considerable geographical variation in CRC incidence and outcome. Population screening has been introduced in many high-incidence countries^2^ and has been shown to decrease CRC mortality^3–6^. However, there are significant intercountry variations in the demographics of the screened population, CRC detection rate, and method of bowel screening^7^. Despite the premise that screening aims to detect CRC in asymptomatic individuals, the majority of incident cancers are diagnosed in patients presenting with symptoms in countries with implementation of screening^8,9^.

Although a minority of population bowel cancer screening programmes use only colonoscopy^7^, most programmes deploy a two-stage approach, with a faecal immunochemical test (FIT) for occult blood in the stool used as the initial screen to determine access to investigation, which is usually colonoscopy. The quantifiable nature of FIT allows bowel screening programmes to set bespoke thresholds for further investigation, aligned with capacity for colonoscopy in the relevant healthcare system. As a result, FIT thresholds for bowel screening programmes range worldwide from 15 to 150 µg haemoglobin (Hb) per g. Hence, there is considerable variation in detection of early-stage lesions, which bleed less^10^.

The bowel screening FIT threshold employed directly affects the interval cancer rate, defined as CRC diagnosed between screening rounds following a negative bowel screening result. The prevalence of interval CRC (I-CRC) ranges from 1.8 to 9.0%, with such tumours more likely to be located in the proximal colon^11^. Furthermore, women are more likely to be diagnosed with an I-CRC^12–14^, highlighting an unintended sex-based health inequality. To counter this, some programmes operate different FIT thresholds for men and women^15^.

This study aimed to determine the rate of I-CRCs within one screening region of Scotland (National Health Service (NHS) Lothian, population approximately 916 000), and the relationship with stage, time to diagnosis, and survival. A further aim was to identify risk factors associated with interval cancers as a basis to explore mechanisms to detect CRC earlier.

## Methods

Regional (NHS Lothian) data from participation in the Scottish Bowel Screening Programme (SBoSP), from the commencement of FIT screening in November 2017 to October 2021 was retrieved from the SboSP for the analyses (316 583 tests). The SBoSP mails FIT kits (HM-JACKarc^TM^, Minaris Medical, Tokyo, Japan) to all persons aged 50–74 years registered with a general practitioner in the region. Those aged ≥ 75 years can opt in to continue screening. All tests are returned to the Scottish Bowel Screening Laboratory in Dundee for analysis, where a positive result is returned if the faecal Hb level is ≥ 80 µg Hb per g. Those with a positive test result are invited for colorectal investigation within the region. Screening is offered every 2 years.

Complete capture of all CRCs in the region was obtained from the South East Scotland Cancer Network database. Review of the screening history of all CRCs in the region permitted assessment of the current bowel screening performance, I-CRC diagnosis, and a projected calculation of the impact of lowering the positivity threshold on service. Pathological reports from patients with CRC were reviewed and cancers staged according to the American Joint Committee on Cancer (AJCC) system. Socioeconomic deprivation was assessed by means of the Scottish Index of Multiple Deprivation (SIMD) tool, which ranks areas by quintile from most (1) to least (5) deprived. The study was approved by the Western General Hospital Colorectal Research and Audit Committee, and was granted local Caldicott approval for the use of patient identifiable data across NHS health boards in Scotland (ID#23115).

### Outcomes of interest

The primary outcome of interest was the rate of I-CRCs detected during the study period among participants with a minimum follow-up of 24 months, and its relationship with FIT results, clinical variables (sex, age, SIMD, CRC location, and stage), time to diagnosis, and survival. The secondary outcome was the identification of risk factors associated interval cancers.

### Statistical analysis

Mann–Whitney *U* test was used for analysis of non-normally distributed data. Categorical data were assessed with the χ^2^ test or Fisher’s exact test as appropriate. The number needed to investigate (NNI; number of positive tests at a set threshold divided by cases of CRC) was determined at varying positivity thresholds and compared with the percentage of CRCs that would be missed. Kaplan–Meier curves were produced for analysis of 2-year survival data. Analysis was performed using R version 4.0.5 (R Foundation for Statistical Computing, Vienna, Austria) with associated packages, and GraphPad Prism™ (GraphPad Software; San Diego, CA, US)

## Results

### Bowel screening uptake and positivity in regional population

Between November 2017 and October 2021, 212 664 people from NHS Lothian participated in the SBoSP, equating to 71.0% of the eligible population participating at least once across the study period. Two successive rounds were performed by 103 919 (34.7%), giving a total of 316 583 tests completed (*Table S1*). When adjusted for the eligible population, uptake of screening by women was higher than for men (71.9 *versus* 70.0%; *P* < 0.001). The number of participants by socioeconomic status was reflective of the region. Uptake of bowel screening differed significantly between the most and least deprived populations (58.6 *versus* 76.9%; *P* < 0.001).

Overall, the positivity rate at the screening threshold of 80 µg Hb per g was 2.7% (8401 people), and it was significantly greater in men than women (3.1 *versus* 2.2%; *P* < 0.001) (*Fig. S1*). To have an positivity rate equal to that in men at 80 µg Hb per g, the threshold for females would need to be 50 µg Hb per g. At the current threshold of 80 µg Hb per g, older patients were more likely to return positive samples, as were people from more deprived areas.

### CRC diagnoses in screened individuals with positive screening test

To assess CRC diagnosis regionally within the bowel screening programme, screening data were censored to those who participated between November 2017 and December 2020 to provide a minimum follow-up of 24 months (median 45 (interquartile range 38–53) months). The number of people completing 1 round of FIT screening was 189 379, of whom 35 623 completed a subsequent screening round (total 225 002 tests). There were 546 CRCs diagnosed within 2 years of completing bowel screening (*Table 1*). This accounted for 546 (19.5%) of all 2802 CRCs diagnosed regionally during the participation period and 2 years subsequently.

**Table 1:** Demographics of patients diagnosed with CRC within 2 years of bowel screening

	No. of patients* (*n* = 546)
Age (years), median (i.q.r.)	67 (61–71)
**Sex**	
Men	296 (54.2%)
Women	250 (45.8%)
**SIMD**	
5	206 (37.7%)
4	90 (16.5%)
3	83 (15.2%)
2	117 (21.4%)
1	49 (9.0%)
Unknown	1 (0.2%)
**AJCC stage**	
I	159 (29.1%)
II	137 (25.1%)
III	144 (26.4%)
IV	101 (18.5%)
Palliated	5 (0.9%)
**Previous screening FIT (µg Hb per g)**	
≥ 200	225 (41.2%)
150–199	19 (3.5%)
80–149	45 (8.2%)
10–79	121 (22.2%)
< 10	136 (24.9%)

^*^Values are *n* (%) unless otherwise stated. CRC, colorectal cancer; i.q.r., interquartile range; SIMD, Scottish Index of Multiple Deprivation (5, least deprived; 1, most deprived); AJCC, American Joint Committee on Cancer; FIT, faecal immunochemical test; Hb, haemoglobin.

**Fig. 1:**
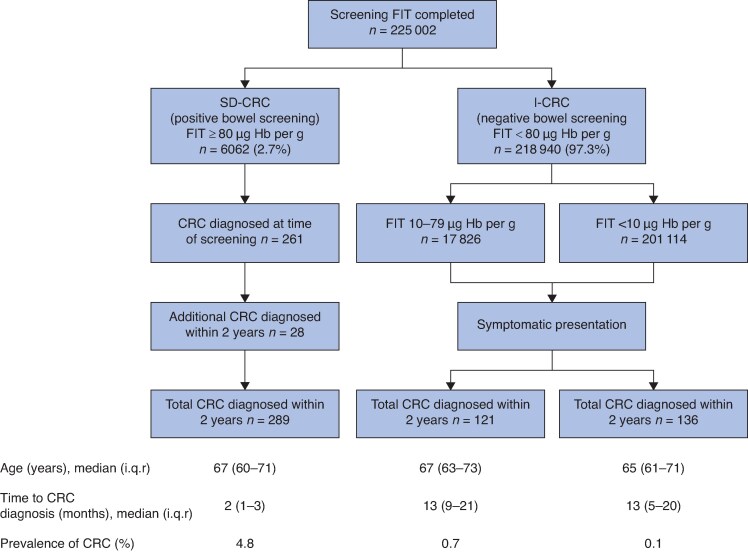
CRC diagnosed in participants of bowel screening A total of 546 patients were diagnosed with colorectal cancer (CRC). FIT, faecal immunochemical test; SD-CRC, screen-detected CRC; Hb, haemoglobin; I-CRC, interval CRC; i.q.r., interquartile range.

Two hundred and eighty-nine CRCs (52.9%) diagnosed within 2 years of screening had a positive bowel screening FIT result of ≥ 80 µg Hb per g (*Fig. 1*). Of these screen-detected CRCs (SD-CRCs), 28 were not diagnosed at the time of screening following a positive FIT, for reasons provided in *Table S2*. Colonoscopy did not detect CRC in 16 patients, equating to an overall miss rate of 5.5% (16 of 289).

### Demographics of I-CRCs compared with SD-CRCs

Some 257 CRCs were defined as I-CRCs as they were detected after a negative screening result and before the next round of screening (*Fig. 1*). The interval cancers accounted for 47.1% of cancers diagnosed in patients participating in bowel screening (257 of 546). Of the I-CRCs, almost half (121 of 257) had detectable occult blood at a level between 10 and 79 µg Hb per g, and CRC was diagnosed at a median of 13 months after screening. Of all CRCs diagnosed in the 2-year period for patients undertaking bowel screening, 24.9% had a screening FIT result below 10 µg Hb per g. The time to diagnosis was substantially longer among patients with I-CRC who had negative bowel screening (median 13 *versus* 2 months; *P* < 0.001); however there was no difference in time to diagnosis between those with a detectable FIT result of 10–79 µg Hb per g and those with a value below 10 µg Hb per g (*P* = 0.138).

Next, the difference in SD-CRCs and I-CRCs in different participant subgroups was explored. Men completed 107 086 of 225 002 tests (47.6%) and accounted for 296 (54.2%) of the CRCs diagnosed. At the bowel screening threshold of 80 µg Hb per g, the I-CRC rate (CRC within 2 years of negative bowel screening) in was 44.6% in men (132 of 296) and 50.0% in women (125 of 250). To reduce the I-CRC rate in women to that in men, the threshold for investigation would have to be lowered to 55 µg Hb per g (*Fig. S2*). However, there was no significant difference in sex between SD-CRCs and I-CRCs (*Table 2*). I-CRCs were more likely to be located in the proximal colon (*P* = 0.019). Between the ages of 55 and 64 years women had the highest increase in the proportion of I-CRCs compared with men and the proportion of I-CRCs was greatest in those from least deprived areas (*Fig. S2*).

**Table 2:** Comparison between SD-CRC and I-CRC

	SD-CRC	I-CRC	% I-CRC	*P*†
All CRCs	289	257	47.1	
**Sex**				0.229
Female	125	125	50.0	
Male	164	132	44.6	
**Age (years)**				
Median (i.q.r.)	67 (60–71)	66 (61–71)		0.684‡
50–54	34	19	35.8	0.631
55–59	22	21	48.8	
60–64	52	53	50.5	
65–69	87	75	46.3	
70–74	63	59	48.4	
≥ 75	31	30	49.2	
**SIMD**				0.404
5	100	106	51.5	
4	48	42	46.7	
3	44	39	47.0	
2	65	52	44.4	
1	31	18	36.7	
**CRC location** *				0.019
Proximal	87	102	54.0	
Distal	202	155	43.4	
**AJCC stage**				< 0.001
Early (I–II)	179	117	39.5	
Late (III–IV)	110	135	55.1	

^*^Proximal: caecum to distal transverse colon; distal: splenic flexure to rectum. CRC, colorectal cancer; SD-CRC, screen-detected CRC; I-CRC, interval CRC; i.q.r., interquartile range; SIMD, Scottish Index of Multiple Deprivation (5, least deprived; 1, most deprived); AJCC, American Join Committee on Cancer. †χ^2^ or Fisher’s exact test, except ‡Mann–Whitney *U* test.

There was no difference in patient demographics, CRC location or stage between the I-CRCs with a screening FIT result < 10 µg *versus* 10–79 µg Hb per g (*Table S3*).

### Stage and survival of I-CRCs

To investigate whether time to diagnosis in patients with a negative bowel screening result (I-CRC) affected clinical outcomes, the stage of CRC at diagnosis was examined (*Table 3*). I-CRCs were more likely to be at an advanced stage (AJCC III–IV) at diagnosis compared with SD-CRCs (53.6 *versus* 38.1%; *P* < 0.001) (*Table 2*). This was true irrespective of whether the FIT level was below the investigation threshold (10–79 µg Hb per g) or below the test positivity threshold used in symptomatic patients (< 10 µg Hb per g). This increased advanced stage of CRC was parallelled by worse overall 3-year survival outcomes in patients who had negative bowel screening and as such had I-CRCs (*Fig. S3*). When patients presented symptomatically there was no significant difference in later-stage disease between those with a FIT result < 10, between 10 and 79, or ≥ 80 µg Hb per g (*Table S4*). The overall 2-year survival was rate was 89.2% for patients with FIT-positive SD-CRCs *versus* 77.0% in those with FIT-negative I-CRCs (*P* < 0.001). When adjusted for early (AJCC I–II)/advanced (III–IV) stage cancer, there was a trend towards better survival for patients with advanced CRCs that were diagnosed by screening. However, among the early-stage cancers the 2-year mortality rate was worse if the CRC was not detected by bowel screening (*P* = 0.020).

**Table 3:** CRC stage of diagnosis and previous bowel screening result in 546 patients

AJCC stage	Bowel screening result (µg Hb per g)			
	≥ 80	< 80	< 10	10–79
All CRCs	289	257	136	121
I	104	55	33	22
II	75	62	30	32
III	73	71	41	30
IV	37	64	30	34
Palliated	0	5	2	3
Advanced CRC*	110 (38.1%)	135 (52.5%)	71 (53.0%)	64 (54.2%)
*P*†	–	< 0.001	0.003	0.004

Values are *n* (%). *American Joint Committee on Cancer (AJCC) stage III–IV. CRC, colorectal cancer; Hb, haemoglobin. †*Versus* ≥ 80µg Hb per g (χ^2^ test); patients who received palliative treatment were excluded from the analysis.

### Optimum FIT threshold to balance colonoscopy caseload against missed cancer

**Fig. 2:**
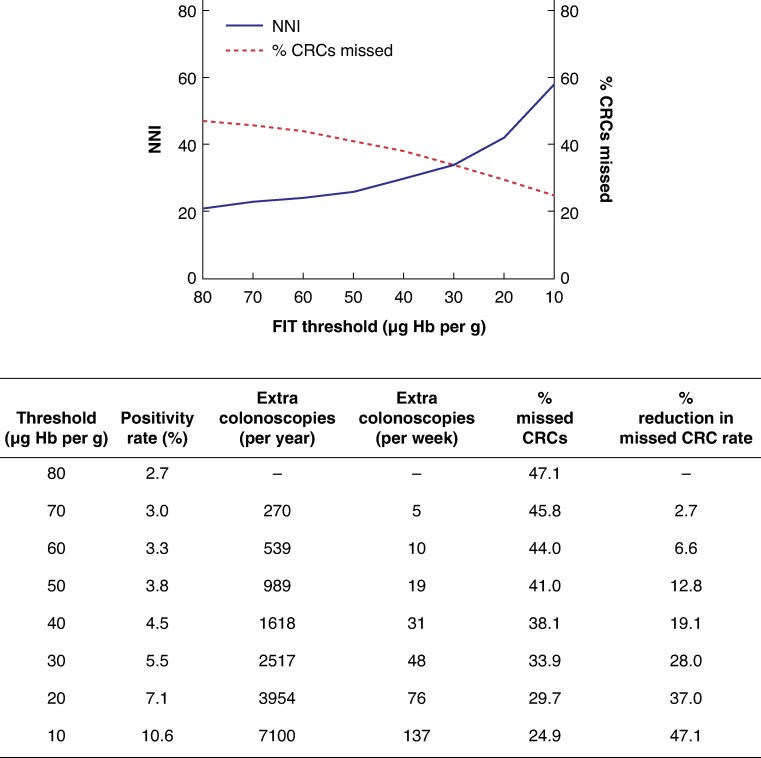
Effect of reducing the bowel cancer screening programme threshold on missed CRC rate and workload CRC, colorectal cancer; NNI, number needed to investigate; FIT, faecal immunochemical test; Hb, haemoglobin.

To investigate the impact of lowering the FIT threshold, the percentage of missed CRCs was compared against the NNI above a threshold to identify one case of CRC (*Fig. 2*). The FIT threshold offering the optimal balance between colonoscopy workload and missed CRC was 30 µg Hb per g. Indeed, reducing the screening threshold to 30 µg Hb per g in the study population would lead to an estimated 28.0% reduction in the missed CRC rate but would require additional capacity of about 48 colonoscopies per week.

## Discussion

This study found that almost half of the patients diagnosed with CRC within 2 years of participating in bowel screening received a negative screening result (threshold < 80 µg Hb per g). Of these, 121 of 257 (47.1%) had detectable blood in their stool at the threshold of 10 µg Hb per g. Patients with a negative bowel screening result were diagnosed once symptoms developed a median of 13 months after the screening test, by which time more cancers were at a later stage, and, overall, 2-year survival was worse. It is possible that patients are falsely reassured by a negative bowel screening result and thus seek medical advice later when symptoms occur. This study has also demonstrated variability in uptake and screening test performance related to sex, age, and socioeconomic deprivation within the resident population of a circumscribed region in Scotland. These inequalities are reflected in the interval cancer rates.

Increasing the uptake of bowel screening remains an important public health issue; as demonstrated in this study, men have a lower rate of uptake than women, and those from lower socioeconomic regions are less likely to participate. Uptake of screening in the region (65%) is comparable to that in the rest of Scotland^16,17^ and the UK^18^. In comparison to other countries that use FIT bowel screening, uptake is very good and close to the leading reported uptake of 71% in the Netherlands^7,19^. In keeping with similar data, women were more likely to participate in bowel screening^20,21^ and, on average, men had a greater FIT result generating a higher positivity rate^22^. Throughout the UK, the introduction of FIT over guaiac fecal occult blood test (gFOBT) has improved this disparity between the sexes^23^, and targeted randomized clinical trials (RCTs) are attempting to close the gap further^24^. Inequalities in CRC outcomes are also associated with lower screening uptake in low socioeconomic groups. Modelling of strategies to increase participation in low socioeconomic groups found annual reinvitation to be most cost-effective^25^. A further large RCT^26^ aims to test the use of completion goals and planning tools for increasing participation.

Bowel screening aims to identify CRCs early to improve survival outcomes, and the UK National Screening Committee^27^ strategy for bowel screening has proposed that the threshold of biennial FIT should be as close as possible to 20 µg Hb per g. Nonetheless, the caveat of feasibility of delivery of additional colonoscopy and pathology workload remains and hence thresholds are determined by service capacity rather than drive to detect cancer early. Indeed, lowering the thresholds first for women, and/or targeting those aged less than 65 years may level the SD-CRC rate. Although efforts to increase screening uptake are laudable and should continue in parallel, these will take longer to translate into patient benefit. Lowering the FIT threshold for screening would result in an immediate increase in earlier detection of CRC among those who engage in the screening programme. It is of particular concern that participants with a detectable FIT result between 10 and 79 µg Hb per g had later-stage disease on eventual presentation, raising the question of being falsely reassured by a negative test. This variation in stage by FIT result is in contrast to local findings in symptomatic patients^28^.

I-CRCs were more likely to be located in the proximal colon and were associated with poorer survival. These key findings have been replicated in a similar FIT bowel screening study from New Zealand^29^. Indeed, anaemia is associated with CRC, and has been associated with CRCs that are FIT-negative^30,31^. In the present study, 47.1% of CRCs diagnosed within 2 years of a negative bowel screening result had a detectable FIT result between 10 and 79 µg Hb per g, with the remainder having no blood detectable in stool.

Improvements to bowel screening programmes could be made by utilizing the quantitative nature of FIT to target these subgroups (FIT 10-79 µg Hb per g, and/or anaemia) for research to establish mechanisms that may enrich early detection. When repeated on a subsequent stool, an individual’s FIT result can change and traverse clinically significant thresholds^31^. Evidence for this in the screening population is mixed, but reductions in missed CRCs have been reported with repeat testing^32^. A sequential screening strategy targeting those with a FIT level of 10–79 µg Hb per g, with either repeat FIT and/or serum full blood count, would explore additional CRC detection and workload pressures *versus* simply lowering the threshold. Further risk stratification for bowel screening participants could be implemented, including the addition of circulating tumour DNA in blood, polygenic single nucleotide polymorphisms or assessment of the faecal microbiome^33^. Although some of these have been explored theoretically, evidence suggests that the additional gain in risk stratification is marginal and, given the more nascent nature of these technologies with limited access, lowering the FIT positivity threshold would be more cost-effective^34,35^.

The present study has shown how a reduction in the screening threshold to 30 µg Hb per g could balance workload and missed CRCs. FIT is used at different thresholds in the symptomatic and screening populations. Currently, for symptomatic patients, the threshold for further investigations is set at 10 µg Hb per g^36^. However, a number of studies^37–40^ have suggested the economic benefits of raising that to between 20 and 40 µg Hb per g. It maybe that the FIT-directed pathways for the diagnosis of screening and symptomatic patients need to be considered together to create the most clinically effective model, and this should be an area of further work. It is important to accept, however, that screening with FIT will not detect all potential I-CRCs as nearly one-quarter had a screening FIT value below 10 µg Hb per g.

This study is limited owing to its single-region nature. Although it used complete capture data from the region, the findings may not be generalizable to other populations in the UK. However, the comparable uptake and positivity rates support the notion that these are representative results. The use of registry data for the detection of CRCs is a strong method for ensuring total capture, but it is not as robust as a design involving prospective follow-up of all patients. The missed cancer rate in patients who did undergo colonoscopy was 5.5%, in keeping with reported literature from the UK^41,42^. The strengths of the study are the description of FIT in SD-CRCs and I-CRCs, and complete capture of the screening population and subsequent CRCs diagnosed.

Overall, inequities exist within the screening programme with women, particularly those aged less than 65 years and from areas of low socioeconomic deprivation, being at increased risk of having I-CRCs. With application of a universal threshold for further diagnostic investigation, this group also has a lower screening positivity rate. There are a substantial number of bowel screening-negative CRCs diagnosed within 2 years of screening. Nearly half of these have detectable FIT levels and could be diagnosed earlier by lowering the diagnostic threshold. Indeed, the programme may also inadvertently generate inequity by reassuring those with detectable blood in stool, resulting in later-stage presentation. Strategies to improve early detection of CRC in those with subscreening thresholds need to be explored with healthcare and public partnerships.

## Supplementary Material

zraf096_Supplementary_Data

## Data Availability

Data available on request.
