# Supplementary material for: Risk factors and clinical consequences of interval cancers arising within faecal immunochemical testing-based colorectal cancer screening programme
Source: BJS Open. 2025 Oct 8;9(5):zraf096. doi: 10.1093/bjsopen/zraf096 (PMC12507088; doi:10.1093/bjsopen/zraf096)
Supplement: zraf096_Supplementary_Data [file zraf096_supplementary_data.docx]

**Risk Factors and Clinical Consequences of Interval Cancers Arising Within FIT-based Bowel Screening Programme**

Authors

Gerrard AD^1,2^, Garau R^1,2^, Maeda Y^1,3^, Thomson A^4^, Theodoratou E^1,5^, Dunlop MG^1,6^, Din FVN^1,2^

^1^ *Cancer Research UK Scotland Centre, Institute of Genetics and Cancer, University of Edinburgh, Edinburgh, UK*

^2^ *Department of Colorectal Surgery, Western General Hospital, Edinburgh, Scotland*

^3^ *Department of Surgery, Queen Elizabeth University Hospital, Glasgow, Scotland*

*^4^ Lothian Analytical Services, NHS Lothian, Edinburgh, Scotland*

^5^ *Centre for Global Health, Usher Institute, The University of Edinburgh, Edinburgh, Scotland*

^6^ *UK Colon Cancer Genetics Group, Medical Research Council Human Genetics Unit, Medical Research Council Institute of Genetics & Cancer, Western General Hospital, The University of Edinburgh, Edinburgh, UK*

**Corresponding author.**

Farhat Din Reader & Honorary Consultant Colorectal Surgeon

Address: Cancer Research UK Scotland Centre, Institute of Genetics and Cancer, University of Edinburgh, Crewe Road, Edinburgh, UK, EH4 2XU

Email: [Farhat.Din@ed.ac.uk](mailto:Farhat.Din@ed.ac.uk)

**Supplementary Materials - Index**

| **Supplementary Tables** |  |
| --- | --- |
| **Supplementary Table 1**  Uptake of bowel screening within the study period by age, sex and SIMD compared with the eligible study population | *Pag. 3* |
| **Supplementary Table 2**  Characteristics of patients with a positive bowel screening result, not diagnosed within the bowel screening programme | *Pag 5* |
| **Supplementary Table 3**  Comparison of I-CRC by screening FIT <10µg Hb/g vs 10-79µg Hb/g | *Pag 6* |
| **Supplementary Table 4**  Local symptomatic CRC staging by FIT category | *Pag 7* |

| **Supplementary Figures** |  |
| --- | --- |
| **Supplementary Figure 1**  Positivity rates of bowel screening results within the region by (A) Sex, (B) Age and (C) Socioeconomic deprivation | *Pag. 8* |
| **Supplementary Figure 2**  Proportion CRCs diagnosed as interval cancer with previously ‘negative’ bowel screening by (A) Sex, (B) Age and (C) Socioeconomic deprivation.  Comparison of I-CRC by screening FIT <10µg Hb/g vs 10-79µg Hb/g | *Pag 9* |
| **Supplementary Figure 3**  (A) All cause two-year survival data and adjusted for (B) Early & (C) Advanced AJCC stage in patients diagnosed following positive bowel (SD-CRC, ≥80µg Hb/g) or negative (I-CRC, <80µg Hb/g). | *Pag 10* |

|  | **NHS Lothian population eligible for screening during study period** | | **Participated at least once in screening *(%)*** | | **Participated in second round of screening *(%)*** |
| --- | --- | --- | --- | --- | --- |
| **Number of people** | | 299,623 | | 212,664 *(71.0)* | 103,919 *(34.7)* |
| Males | | 145,286 | | 101,706 *(70.0)* | 48,916 *(33.7)* |
| Females | | 154,337 | | 110,958 *(71.9)* | 55,003 *(35.6)* |
| **Age (Years)** | |  | |  |  |
| *All persons* | |  | |  |  |
| 50-54 | | 110,790 | | 65,268 *(58.9)* | 17,252 *(15.6)* |
| 55-59 | | 56,170 | | 35,771 *(63.7)* | 18,440 (32.8) |
| 60-64 | | 47,113 | | 37,556 *(79.7)* | 21,749 *(46.2)* |
| 65-69 | | 43,442 | | 39,049 *(89.9)* | 22,108 *(50.9)* |
| 70-74 | | 36,396 | | 25,363 *(69.7)* | 15,972 *(43.9)* |
| 75+ | | 63,990* | | 9,644 *(15.1)* | 8,396 *(13.1)* |
| *Males* | |  | |  |  |
| 50-54 | | 54,485 | | 31,468 *(57.8)* | 8,136 *(14.9)* |
| 55-59 | | 27,520 | | 17,326 *(63.0)* | 8,793 *(32.0)* |
| 60-64 | | 23,040 | | 18,002 *(78.1)* | 10,240 *(44.4)* |
| 65-69 | | 20,731 | | 18,507 *(89.3)* | 10,399 *(50.2)* |
| 70-74 | | 16,964 | | 11,895 *(70.1)* | 7,458 *(44.0)* |
| 75+ | | 25,897* | | 4,502 *(17.4)* | 3,889 *(15.0)* |
| *Females* | |  | |  |  |
| 50-54 | | 56,305 | | 33,800 *(60.0)* | 9,116 *(16.2)* |
| 55-59 | | 28,650 | | 18,445 *(64.4)* | 9,647 *(33.7)* |
| 60-64 | | 24,073 | | 19,554 *(81.2)* | 11,509 *(47.8)* |
| 65-69 | | 22,711 | | 20,542 *(90.4)* | 11,709 *(51.6)* |
| 70-74 | | 19,432 | | 13,468 *(69.3)* | 8,514 *(43.8)* |
| 75+ | | 38,093* | | 5,142 *(13.5)* | 4,507 *(11.8)* |
| **SIMD** | |  | |  |  |
| *All persons* | |  | |  |  |
| 5 | | 102,104 | | 78,473 *(76.9)* | 40,583 *(39.7)* |
| 4 | | 54,469 | | 40,510 *(74.4)* | 20,244 *(37.2)* |
| 3 | | 51,304 | | 34,326 *(66.9)* | 16,595 *(32.3)* |
| 2 | | 61,600 | | 37,784 *(61.3)* | 17,650 *(28.7)* |
| 1 | | 30,146 | | 17,658 *(58.6)* | 7,692 *(25.5)* |
| *Males* | |  | |  |  |
| 5 | | 49,334 | | 36,807 *(74.6)* | 18,775 *(38.1)* |
| 4 | | 26,618 | | 19,409 *(72.9)* | 9,523 *(35.8)* |
| 3 | | 25,027 | | 16,494 *(65.9)* | 7,925 *(31.7)* |
| 2 | | 29,609 | | 17,946 *(60.6)* | 8,273 *(27.9)* |
| 1 | | 14,698 | | 8,706 *(59.2)* | 3,721 *(25.3)* |
| *Females* | |  | |  |  |
| 5 | | 52,770 | | 41,666 *(79.0)* | 21,808 *(41.3)* |
| 4 | | 27851 | | 21,101 *(75.8)* | 10,721 *(38.5)* |
| 3 | | 26,277 | | 17,832 *(67.9)* | 8,670 *(33.0)* |
| 2 | | 31,991 | | 19,838 *(62.0)* | 9,377 *(29.3)* |
| 1 | | 15,448 | | 8,952 *(57.9)* | 3,971 *(25.7)* |

Supplementary Table 1: Uptake of bowel screening within the study period by age, sex and SIMD compared with the eligible study population

*Persons age 75 and over not routinely invited to screening. SIMD; Scottish Index of Multiple Deprivation (5: least deprived, 1: most deprived). Eligible population as per mid-year population statistics.

Supplementary Table 2: Characteristics of patients with a positive bowel screening result, not diagnosed within the bowel screening programme

Supplementary Table 3: Comparison of I-CRC by screening FIT <10µg Hb/g vs 10-79µg Hb/g

|  | **Screening FIT Result (µg Hb/g)** | |  |
| --- | --- | --- | --- |
|  | **<10** | **10-79** | **p-value** |
| Total CRCs | 136 | 121 |  |
| Sex *(%)* |  |  |  |
| Female | 69 *(50.7)* | 56 *(46.2)* |  |
| Male | 67 *(49.3)* | 65 *(53.7)* | 0.532 |
| Age (Years) |  |  |  |
| Median (IQR) | 65 (61-71) | 67 (63-73) | 0.192 |
| 50-54 | 9 *(6.6)* | 10 *(8.3)* |  |
| 55-59 | 15 *(11.0)* | 6 *(5.0)* |  |
| 60-64 | 31 *(22.8)* | 22 *(18.2)* |  |
| 65-69 | 39 (28.7) | 36 *(29.8)* |  |
| 70-74 | 25 *(18.4)* | 34 *(28.1)* |  |
| 75+ | 17 (12.5) | 13 *(10.7)* | 0.255 |
| SIMD *(%)* |  |  |  |
| 5 | 59 *(43.4)* | 47 *(38.8)* |  |
| 4 | 22 *(16.2)* | 20 *(16.5)* |  |
| 3 | 25 *(18.4)* | 14 *(11.6)* |  |
| 2 | 23 *(16.9)* | 29 *(24.0)* |  |
| 1 | 7 *(5.1)* | 11 *(9.1)* | 0.265 |
| CRC Location *(%)* |  |  |  |
| Proximal | 51 *(37.5)* | 51 *(42.1)* |  |
| Distal | 85 *(62.5)* | 70 *(57.9)* | 0.523 |
| CRC Stage *(%)* |  |  |  |
| Early | 63 *(47.0)* | 54 *(45.8)* |  |
| Late | 71 *(53.0)* | 64 *(54.2)* | 0.900 |

2 cases of <10, and 3 cases of 10-79 palliated without formal AJCC staging

Supplementary Table 4a: Local symptomatic CRC staging by FIT category

|  |  | **FIT (µg Hb/g)** | | |
| --- | --- | --- | --- | --- |
|  |  | <10 | 10-79 | ≧80 |
| **AJCC Stage** | Early (1&2) | 9 (52.9%) | 17 (44.7%) | 64 (43.0%) |
|  | Late (3&4) | 8 (47.1%) | 21 (55.3%) | 85 (57.0%) |

Supplementary Table 4b: Local symptomatic CRC staging by FIT category

| Comparison | p-value |
| --- | --- |
| <10 : 10-79 | 0.771 |
| <10 : ≧80 | 0.451 |
| 10-79 : ≧80 | 0.856 |

Supplementary Figure 1: Positivity rates of bowel screening results within the region by (A) Sex, (B) Age and (C) Socioeconomic deprivation

SIMD; Scottish Index of Multiple Deprivation (5: least deprived, 1: most deprived)

Supplementary Figure 2: Proportion CRCs diagnosed as interval cancer with previously ‘negative’ bowel screening by (A) Sex, (B) Age and (C) Socioeconomic deprivation.

SIMD; Scottish Index of Multiple Deprivation (5: least deprived, 1: most deprived)

Supplementary Figure 3: (A) All cause two-year survival data and adjusted for (B) Early & (C) Advanced AJCC stage in patients diagnosed following positive bowel (SD-CRC, ≥80µg Hb/g) or negative (I-CRC, <80µg Hb/g).

A. Overall all -cause mortality

B. Early (AJCC stage 1&2) all-cause mortality

C. Late (AJCC stage 3&4) all-cause mortality
